# Sentinel-lymph-node procedures in early stage cervical cancer: a systematic review and meta-analysis

**DOI:** 10.1007/s12032-014-0385-x

**Published:** 2014-11-28

**Authors:** Xiao-juan Wang, Fang Fang, Ye-fei Li

**Affiliations:** 1 Obstetrics and Gynecology Hospital of Fudan University, 419 Fangxie Road, Shanghai, 200011 People’s Republic of China; 2 Cancer Hospital of Guangxi Medical University, 71 Hedi Road, Nanning, 500021 People’s Republic of China

**Keywords:** Sentinel-lymph-node, Early cervical cancer, Meta-analysis, Nodal metastases

## Abstract

We performed a meta-analysis to assess the accuracy of sentinel-lymph-node (SLN) procedures for the assessment of nodal metastases in patients with early stage cervical cancer. Studies of SLN procedures for detecting nodal metastases in patients with early stage cervical cancer were systematically searched in MEDLINE and EMBASE between January 1, 2000 and August 30, 2013. We identified 49 eligible studies, which included 2,476 SLN procedures. The mean overall weighted-detection rate was 0.93 (95 % CI 0.92–0.94), at a pooled sensitivity of 0.88 (95 % CI 0.84–0.90) with limited heterogeneity (*χ* ^2^ = 80.57, degrees of freedom = 47, *p* = 0.002). Subgroup analysis of sensitivity and the rate of detection of different tracer techniques and surgery methods used in conjunction with an SLN procedures were as follows: studies using combined techniques, 0.88 (95 % CI 0.84–0.91) and 0.97 (95 % CI 0.96–0.98); studies using metastable technetium-99, 0.87 (95 % CI 0.78–0.93) and 0.90 (95 % CI 0.87–0.93); studies using blue dye, 0.87 (95 % CI 0.79–0.93) and 0.87 (95 % CI 0.84–0.90); studies using laparotomy, 0.86 (95 % CI 0.80–0.90) and 0.87 (95 % CI 0.83–0.91); studies using laparoscopy, 0.90 (95 % CI 0.86–0.94) and 0.93 (95 % CI 0.90–0.96); and studies using robot-assisted surgery, 0.84 (95 % CI 0.72–0.92) and 0.92 (95 % CI 0.88–0.95). We concluded that the SLN procedure performs well diagnostically for the assessment of nodal metastases in patients with early stage cervical cancer.

## Introduction

Cervical cancer is the third most commonly diagnosed cancer and the fourth leading cause of cancer death in women worldwide with most cases occurring in developing countries [1]. Even with dramatic treatment changes, increased incidence and deaths due to cervical cancer continues to be a problem: 12,340 new cases and 4,030 deaths occurred in 2013, from 11,150 to 3,670 in 2007 in the USA [2, 3]. Cervical cancer diagnosis is made by cervical biopsy or conization. Although cervical screening is excellent for cancer prevention, this disease continues to be diagnosed in locally advanced stages. The Federation Internationale de Gynecologie et d’Obstetrique (FIGO) clinical staging system does not include evaluation of lymph node involvement, although lymph node metastasis is an important prognostic factor in cervical cancer in addition to parametrial cancer extension and positive surgical margins [4].

Radical hysterectomy with pelvic lymphadenectomy is the standard treatment for early stage cervical cancer. However, pelvic lymph node metastases are detected in 0–4.8 and 0–17 % of patients with stage IA and IB cervical cancer, respectively [5]. Moreover, lymph node involvement is observed in only 12–27 and 25–29 % of patients with stages IIA and IIB cervical cancer, respectively [6, 7], suggesting that about three quarters of all patients routinely received pelvic lymph node dissection despite the absence of metastasis. Lymph node dissection not only increases operative time and blood loss but also causes occasional leg lymphedema and nerve and blood vessel injuries [8]. Moreover, removal of “healthy” lymph nodes may negatively influence the immune system. Thus, in most patients with cervical cancer, lymph node dissection could be omitted.

Cabanas reported the existence of a so-called sentinel-lymph-node (SLN), confirming that this site was the first location of metastasis and that in clinically non-suspicious nodes, this node was frequently the only one affected [9]. When nodal metastases occur, the SLN will be initially involved and as such SLN biopsy has been implemented in the standard of care for patients with melanoma and breast cancer [9, 10]. If the SLN concept is valid in cervical cancer, most patients with early stage disease could avoid pelvic lymphadenectomy by both preoperative radioisotope injection of Tc99m or intraoperative blue dye with a confirmation of a metastasis-free SLN status. Because current studies offer inconclusive data about this procedure, we performed a meta-analysis to evaluate the diagnostic performance of SLN procedures with respect to sensitivity and early stage cervical cancer detection.

## Materials and methods

### Search strategy and selection criteria

A comprehensive systematic search for published studies was performed independently by Wang and Li from January 1, 2000 to August 31, 2013, using Embase and PubMed databases. Predefined search terms were used to identify reports about the diagnostic performance of the SLN procedure in patients with early cervical cancer. We used a search algorithm that was based on a combination of text words: “sentinel-lymph-node” AND “cervical cancer”. Review articles, letters, comments, conference proceedings, unpublished data and case-reports were not selected for our study. We included studies meeting the following inclusion criteria: enrollment of at least 12 patients; prospective design to assess effectiveness of identification and diagnostic performance of the SLN procedure; most (>80 %) enrolled patients had early stage cervical cancer (FIGO I-IIA) and reported positivity rates (ie, a clearly described histopathological analysis and specimen-handling procedure). To avoid overlapping patient data in duplicate publications, we included the more recent articles with the largest sample size.

### Reference standard and test results

The approach of this report differs from similar studies, because, in line with common clinical practice, the false-positivity rate was, by definition, zero. When the SLN is the only positive node identified, the SLN procedure is considered to be successful. The detection rate was calculated by the number of procedures in which at least one SLN was identified, divided by the total number of procedures undertaken. The sensitivity of the SLN procedure was defined as the number of true positives in patients with positive histopathological findings (true positives/[true positives + false negatives]). A true positive SLN was defined as a positive SLN identified with histopathological techniques (hematoxylin and eosin staining, serial sectioning, immunohistochemistry, or RT-PCR), independent of regional lymph node status. Sentinel-lymph-node procedure yielding tumor-negative sentinel node(s) in combination with tumor-positive non-sentinel nodes were classified as false negative.

### Data extraction

Wang and Li extracted relevant data from all full-text publications using a standardized data abstraction form. They were blinded to the identity of study investigators and institution. The data extraction form was comprised of the following items: year of publication and origin, histopathological technique used, number of patients included, type of study design (prospective, retrospective, or unknown), and method of SLN identification (radiotracer or dye, method, and mode of injection).

To assess the quality and applicability of the studies included in this report, we used an established quality-rating system for diagnostic studies based on QUADAS [11]. Wang and Li independently reviewed each article to extract relevant study characteristics and results using a standard form. We divided the criteria list into two subgroups: internal and external validity. The criteria could be scored as “yes,” “no,” or “not mentioned” in the publication. The internal validity items focused on the validity of the reference test (histology), consecutive patients, blinded interpretation of pathological results, and prospective studies. The external validity items focus on stage of disease (FIGO), the type of patient population and spectrum, demographics, the inclusion/exclusion criteria, detection of sentinel node (SN) technique, localization of SN (and/or bilateral SN) described, scintigraphy, and description of SN criteria.

### Statistical analysis

The sensitivity of the SN procedure was determined from the number of true positive (TP) and false-negative (FN) results from the 2 × 2 contingency table of the individual studies. Studies that did not present patients with tumor-positive sentinel node were excluded from statistical pooling of sensitivity but were included for pooling of the SN detection rate. The detection rate was defined as the percentage of procedures in which at least one sentinel node was identifiable. Because false-positive SLN procedures are impossible in our context, we set the specificity of the procedure at 100 %. Therefore, sensitivity was pooled with a random-effect analysis, without taking specificity into account. Potential heterogeneity of sensitivity results was analyzed with the Chi-squared test. We performed a subgroup analysis for the three SN detection techniques: Tc99m, blue dye, and the combination of both. Detection rates and sensitivity for studies using a laparotomic procedure versus studies using a laparoscopic procedure versus studies using a robot-assisted procedure were calculated. Pooled data are presented with 95 % confidence intervals (95 % CI). All data were processed with the metan procedure in STATA version 11.0 (Stata Corp; Texas, USA) and Meta-DiSc version 1.4 (XI Cochrane Colloquium; Barcelona, Spain). In all analyses, a *p* value of 0.05 or less indicated statistical significance.

## Results

We identified 49 eligible studies (Table 1) [6, 7, 12–58]. Two studies [59, 60] were excluded because of insufficient cases, and eight studies were excluded because of duplicate publication [61–68]. Most patients were diagnosed as having early stage cervical cancer (FIGO I–IIA), but one study was excluded because more than 50 % patients had stage IIb cancer [69]. Overall, 2,476 SLN procedures were included as eligible reports, and there were 39 eligible studies [6, 7, 13, 15, 16, 20–22, 26, 28–58] that met at least three of the four internal validity criteria (see Table 2). All studies used a valid reference test (histology) and had a prospective design. Both inclusion and exclusion criteria were offered in 27 studies [15, 26–37, 41–46, 49–54, 56, 57] (see Table 2), but the definition of a SLN varied among studies (variously defined as “blue,” “hot,” or “blue and hot”).

**Table 1:** Characteristics of studies in the meta-analysis

Authors	Year	Origin	Technique	Cases (N)	Surgical method	Tracer	Mode	SN definition	True +	True −	False −	Prevalence
O’Boyle [6]	2000	USA	Serial section, no description	20	LAR	Isosulfan blue dye	4 ml, 4 quadrants	Obvious blue nodes	3	8	1	4/12
Malur [7]	2001	Germany	H&E	50	LAP(45), LAR(5)	Patent blue(9), 99mmtc both(20)	NR, 50 MBq in 1 ml(21)	Audible signals; blue-stained lymph node	5	33	1	6/39
Lantzsch [13]	2001	Austria	Serial section, H&E, IHC SLN only	14	LAR	99mmtc colloid	1 depot, 60–111 MBq	Radioactive nodes	1	12	0	1/13
Rhim [14]	2002	Korea	3 sections, H&E	26	LAR	99mmtc colloid, blue dye	2–3 depot, 10–20 MBq	Blue-stained node; radioactive nodes	4	20	1	5/25
Levenback [15]	2002	USA	Serial sectioning, H&E, IHC Only on SN	39	LAR	Isosulfan blue dye, Tc99m	4 ml 4 quadrant, 1–1.5 ml	Sentinel nodes labeled as blue, hot(radioactive), or both	7	25	1	8/33
Lambaudie [16]	2003	France	Frozen section, IHC only on SLN	12	LAP	Patent blue, 99mmtc colloid	4 ml 4 quadrant, 74 MBq	Blue and/or radioactive sentinel node	2	9	0	2/11
Dargent [17]	2003	France	Serial sections, IHC	70	LAR	Blue dye, Tc99m	2 ml, 1 mCi	Blue-stained/radioactive lymph node	9	110	10	19/129
Van Dam [18]	2003	Belgium	5-mm, sections, H&E, IHC only on SN	25	2 LAP23 LAR	Tc99m	2 quadrant, 60 MBq	Radioactive nodes	5	16	0	5/21
Hubalewska [19]	2003	Poland	H&E, IHC only on SN	37	LAR	Patent blue dye, Tc99m	4 ml, 4 quadrant, 100 MBq	Radioactive nodes, blue-stained nodes	5	30	0	5/35
Niikura [20]	2004	Japan	H&E, IHC on all nodes	20	LAR	Patent blue dye, 99m Tc	4 ml 4 quadrant,38–70 MBq	Radioactive and blue nodes	2	16	0	2/18
Lin Bin [21]	2004	China	Pathological examination	28	LAR	Tc99m	37 MBq	Radioactive nodes	6	21	0	6/21
Martı´nez-Palones [22]	2004	Spain	0.2-mm interval, H&E, IHC only on SN	25	7LAP, 18 LAR	Blue dye, Tc99m	2–4 ml, 20 MBq	Blue-stained nodes	3	19	0	3/23
Pijpers [23]	2004	The Netherlands	Frozen section, 250-μm intervals H&E, IHC only on SN	34	LAP	Patent blue dye, Tc99m	2–4 ml, 4 quadrant, 228 MBq	Blue as well as radioactive nodes	11	21	1	12/33
Marchiole [24]	2004	France	Frozen sectioning, 200-μm intervals, H&E, IHC on all nodes	29	LAP	Patent blue dye	4 ml, 4 quadrant	Unknown	5	21	3	8/29
Di Stefano [25]	2005	Italy	200-μm serial sections, H&E, IHC only on SN	50	LAR	Methylene blue dye	2 ml, 4 quadrant	Blue-stained lymph nodes	9	35	1	10/45
Gil-Moreno [26]	2005	Spain	0.2 mm sections, H&E, IHC only on SN	12	LAP	99mmtc colloid, blue dye	2–4 ml 4 quadrant, 40 MBq	Blue, hot lymph nodes	0	12	0	0/12
Rob [27]	2005	Czech Republic	Frozen section, 40-μm intervals, H&E, IHC only on SN	183	39 LAP,144 LAR	Patent blue dye, Tc99m	2 ml 4 quadrant, 20 MBq	Blue nodes, radioactive nodes	35	124	1	36/160
Angioli [28]	2005	Italy	Serial sectioning, H&E, IHC only on SN	33	LAP	Tc99m colloid	4 quadrant, 60 MBq	Radioactive nodes	6	20	0	6/26
Roca [29]	2005	Spain	0.2 mm sections, H&E, IHC on all nodes	40	12 LAP 28 LAR	Blue dye, Tc99m	2–4 ml 4 quadrant, 74 MBq	Blue-stained nodes	4	36	0	4/36
Sliva [30]	2005	Brazil	2–3 mm intervals H&E, IHC only on SN	56	LAR	Tc99m	4 quadrant, 55–74 MBq	Radioactive nodes	17	32	3	20/52
Lin [31]	2005	Taiwan	250-μm intervals H&E, IHC only on SN	30	LAR	99mmtc colloid	4 quadrant, 202 MBq	Radioactive nodes	7	23	0	7/30
Wydar [32]	2006	Poland	250-μm intervals H&E, IHC only on SN	100	LAR	99mmtc, blue dye	1 ml, 4 quadrant, 35–70 MBq, 4 ml	Radioactive nodes; blue-stained nodes	19	63	3	22/85
Wang [33]	2006	China	H&E, IHC RT-PCR	46	LAP	Methylene blue	2 ml	Blue-stained lymph nodes	28	18	0	28/46
Altgassen [34]	2007	Germany	Frozen	60	LAR	Paten blue	4 ml, 4 quarant, 2 ml with 8 ml NaCl	Blue-stained nodes	11	41	4	15/56
Bats [35]	2007	France	250-μm intervals H&E, IHC on all nodes	25	LAP	Tc99m, patent blue	4 quadrants, 120 MBq, 2 ml	Blue/radioactive nodes	2	21	0	2/23
Kushner [36]	2007	USA	Frozen section, H&E, IHC only on negative nodes	20	LAP	Tc99m, blue dye	1 ml, 4 quadrants, 4 ml	Radioactive nodes, blue-stained nodes	2	18	0	2/20
Darai [37]	2007	France	3 mm interval, H&E, IHC only on SN	54	LAP	Tc99m	4 quadrant, 20 MBq	Blue-stained or hot nodes	11	28	6	17/45
Yong Seok Lee [38]	2007	Korea	Frozen biopsy, H&E	57	LAR	Isosulfan blue And Tc99m	1.0 ml, 10–20 MBq	Blue and/or “hot” nodes	10	46	1	11/57
Seok Ju Seong [39]	2007	Korea	Frozen section, H&E staining	89	LAR	Isosulfan blue dye	1 ml	Blue-stained nodes	10	40	1	11/51
Song-Hua Yuan [40]	2007	China	H&E, IHC only on SN	81	LAR	Methylene blue dye, patent Blue	2–4 ml, 4 ml	Blue-stained nodes	10	51/3	3	13/64
Bats [41]	2008	France	3 mm interval 150-μm, H&E, IHC only on SN	71	LAP	Tc99m; patent blue	0.2 ml, 80 MBq, 4 quadrants	Blue and/or radioactive nodes	16	42	2	18/60
Fader [42]	2008	USA	Imprint cytology and frozen section; H&E, IHC	38	11 LAP, 27 LAR	Tc99m, isosulfan blue	0.5 mCi, 4 quadrants, 1.5 ml	Radioactive nodes, blue-stained nodes	5	29	1	6/35
Kara [43]	2008	Turkey	H&E, IHC	32	LAP	Tc99m, blue dye	4 quadrants, 74 MBq, 4 ml	Radioactive nodes, blue-stained nodes	9	23	0	9/32
Pluta [44]	2009	Czech Republic	Frozen section, H&E, IHC	60	LAR	Patent blue dye, Tc99m	20 MBq, 4 ml	Blue-colored/radioactive nodes	5	55	0	5/60
Vieira [45]	2009	Brazil	Frozen section, H&E on all nodes	56	LAR	Tc99m patent blue dye	600–800 μCi, 4 quadrants, 4 ml	Stained (blue), ‘‘hot’’ (radioactive) or both	5	41	1	6/47
Gortzak-Uzan [46]	2010	Canada	Frozen sections, H&E, IHC only on SN	87	LAP	Tc99m and/or blue dye	0.1–0.2 μCi, 4 quadrants, 4 ml	Blue/hot lymph nodes	14	67	0	14/81
Darlin [47]	2010	Sweden	Frozen section, H&E	105	91 robot-assisted LAP,8 LAP, 6 LAR	Tc99m	1–1.5 ml, 120 MBq, 4 quadrants	Radioactive nodes	17	76	1	18/94
Ogawa [48]	2010	Japan	H&E	82	LAP	Tc99m	4 quadrants, 148 MBq	Radioactive hot nodes	12	60	0	12/72
FOTIOU [49]	2010	Greece	standard and enhanced pathological analysis	42	LAR	Tc99m, blue dye	0.8 ml, 4 quadrants, 2 ml	Radioactive nodes; blue-stained nodes	4	33/1	1	5/38
Cormier [50]	2011	US	Frozen section, H&E,IHC	122	37 LAP or robotic-assisted LAP, 85 LAR	Blue dye, Tc99m	4 ml, unknown	Blue/hot nodes	21	90	3	24/90
Diaz-Feijoo [51]	2011	Spain	H&E, IHC only on negative SLN	22	20 LAP,2 LAR	Tc99m, blue dye	4 quadrants, 144 MBq, less than 1 ml	Nodes as radioactive, blue positive or both blue and hot	4	18	0	4/22
Lecuru [52]	2011	France	200-μm section, H&E, IHC only on negative nodes	139	LAP	Tc99m, patent blue	4 quadrants, 120 MBq, 2 ml	Blue-stained and/or radioactive lymph nodes	23	111	2	25/136
Roy [53]	2011	Canada	Serial section, H&E, IHC only on SN	211	LAP	Blue dye, Tc99m	1 ml, 37 MBq	Blue and/or hot nodes	29	177	3	32/209
Xue-lian Du [54]	2011	China	Frozen section, IHC on all nodes	68	LAP	Tc99m	2.5 ml, 4 quadrants, 100 MBq	Radioactive hot nodes	8	56	0	8/64
Niikura [55]	2012	Japan	Frozen section, H&E, IHC	35	LAP	Tc99m, blue dye	0.4 ml, 60 MBq, 4 quadrants, 4 ml	Radioactive and blue nodes	8	24	3	11/35
Devaja [56]	2012	United Kingdom	H&E, IHC only on positive nodes	86	LAP, LAR	Tc99m, methylene blue dye	4 quadrants, 40 MBq, 4 ml	Blue-stained nodes	9	75	0	9/86
Frumovitz [57]	2012	USA	250-μm intervals, H&E, IHC	20	LAR, LAP, or robotic-assisted LAP	Tc99m, India ink	2 ml, 4 quadrants, 4 ml	Radioactive/blue nodes	3	12	2	5/17
Schaafsma [58]	2012	The Netherlands	H&E, IHC on SN and suspected nodes	18	LAR	Indocyanine green	1.6 ml, 4 quadrants	Nodes with fluorescent hotspots	5	8	1	6/14
Hoogendam [59]	2013	The Netherlands	Frozen section	62	Conventional and robot-assisted LAP	Tc99m, patent blue dye	220–290 MBq, 4 quadrants, 2–4 ml	Blue-stained/radioactive nodes	7	52	3	10/62

*SLN* sentinel lymph node, *H&E* hematoxylin and eosin staining, *IHC* immunohistochemistry, *LAR* laparotomy, *LAP* laparoscopy, *NR* not reported

**Table 2:** Quality rating of included studies

References	Internal validity				External validity								
	Valid reference test	Consecutive patient selection	Blinded interpretation of results	Prospective study	Disease stage	Disease spectrum	Demographics	Inclusion criteria	Exclusion criteria	Detection technique	SN criteria	Scintigraphy	SN localization described
O’Boyle [6]	+	+	−	+	+	+	+	−	−	+	+	+	+
Malur [7]	+	−	−	+	+	+	+	+	+	+	+	+	+
Lantzsch [13]	+	+	−	+	+	+	+	−	−	+	+	+	+
Rhim [14]	+	+	−	+	+	NM	+	−	−	+	−	+	−
Levenback [15]	+	−	−	+	+	+	+	−	−	+	+	+	+
Lambaudie [16]	+	−	−	+	+	+	+	−	−	+	+	+	+
Dargent [17]	+	+	−	−	+	+	−	−	−	+	−	−	+
Van Dam [18]	+	+	−	+	+	88 %	+	+	−	+	+	+	+
Hubalewska [19]	+	−	−	+	+	+	−	+	−	−	+	+	−
Niikura [20]	+	+	−	+	+	+	+	−	−	+	+	+	+
Lin Bin [21]	+	+	−	−	+	+	+	−	−	+	+	+	+
Martı´nez-Palones [22]	+	+	−	+	+	+	+	−	−	+	+	+	+
Pijpers [23]	+	−	−	+	+	+	+	−	−	+	+	+	+
Marchiole [24]	+	+	−	−	+	+	+	−	−	+	+	+	+
Di Stefano [25]	+	+	−	+	+	+	+	+	−	+	+	NRTU	+
Gil-Moreno [26]	+	−	−	−	+	+	+	+	+	+	+	+	−
Rob [27]	+	+	−	+	+	+	+	+	+	+	+	+	+
Angioli [28]	+	+	−	+	+	+	+	+	+	+	+	+	+
Roca [29]	+	+	−	+	+	+	+	+	+	+	+	+	−
Sliva [30]	+	+	−	+	+	+	+	+	+	+	+	+	+
Lin [31]	+	+	−	+	+	+	+	+	+	+	+	+	−
Wydar [32]	+	+	−	+	+	+	+	+	+	+	+	+	+
Wang [33]	+	+	−	+	+	+	+	+	−	−	+	−	−
Altgassen [34]	+	+	−	+	+	85 %	−	+	+	+	−	+	+
Bats [35]	+	+	−	+	+	+	+	+	+	+	+	+	+
Kushner [36]	+	+	−	+	+	+	+	+	+	+	+	+	+
Darai [37]	+	+	−	+	+	+	+	+	+	+	+	+	+
Yong Seok Lee [38]	+	+	−	+	+	+	+	−	−	+	+	−	−
Seok Ju Seong [39]	+	+	−	+	+	+	+	−	−	+	+	−	+
Song-Hua Yuan [40]	+	+	−	+	+	+	+	−	−	+	+	+	+
Bats [41]	+	+	−	+	+	80 %	+	+	+	+	+	+	+
Fader [42]	+	+	−	+	+	+	+	+	+	+	+	+	+
Kara [43]	+	+	−	+	+	+	+	+	+	+	+	+	+
Pluta [44]	+	+	−	+	+	+	+	+	+	+	+	+	+
Vieira [45]	+	+	−	+	+	+	+	+	+	+	+	+	+
Gortzak-Uzan [46]	+	+	−	+	+	+	+	+	+	+	+	+	+
Darlin [47]	+	+	−	+	+	+	+	−	−	+	+	+	+
Ogawa [48]	+	+	−	+	+	+	+	−	−	+	+	+	+
FOTIOU [49]	+	+	−	+	+	+	+	−	−	+	+	+	+
Cormier [50]	+	+	−	+	+	+	+	+	+	+	+	+	+
Diaz-Feijoo [51]	+	+	−	+	+	+	+	+	+	+	+	+	+
Lecuru [52]	+	+	−	+	+	+	+	+	+	+	+	+	+
Roy [53]	+	+	−	+	+	+	+	+	+	+	+	+	+
Xue-lian Du [54]	+	+	−	+	+	+	+	+	+	+	+	+	+
Niikura [55]	+	+	−	+	+	+	+	−	−	+	+	+	+
Devaja [56]	+	+	−	+	+	+	+	−	−	+	+	+	+
Frumovitz [57]	+	+	−	+	+	+	+	+	+	+	+	+	+
Schaafsma [58]	+	+	−	+	+	+	+	+	+	+	+	+	+
Hoogendam [59]	+	+	−	−	+	+	+	−	−	−	−	−	−

+: yes; −: no; *NRTU* no radioactive tracer used, *NM* not mentioned

Blue tracer dye varied from 0.2 to 4 ml [13, 41], and Tc99m activity (MBq) varied from 10 to 290 MBq among studies [38, 59]. In 36 studies [13, 17, 18, 21, 22, 24–27, 30–37, 41–48, 50–52, 54–59], the four-quadrant method was used and four studies [14, 16, 20, 23] did not mention the number of peritumoral injections. In most studies [7, 14, 17–21, 24, 26–33, 36, 37, 40–46, 49–53, 55–58], immunohistochemical SNL staining was performed with hematoxylin and eosin (H&E) and this was negative. SLN sectioning was not uniformly undertaken across studies, but serial sectioning was used in most papers [7, 14, 17, 19, 20, 24–33, 35, 37, 41, 52, 53, 56] ranging from 0.2 to 5 mm sections [7, 20]. SLN identification was classified as in vivo or ex vivo.

**Fig. 1:**
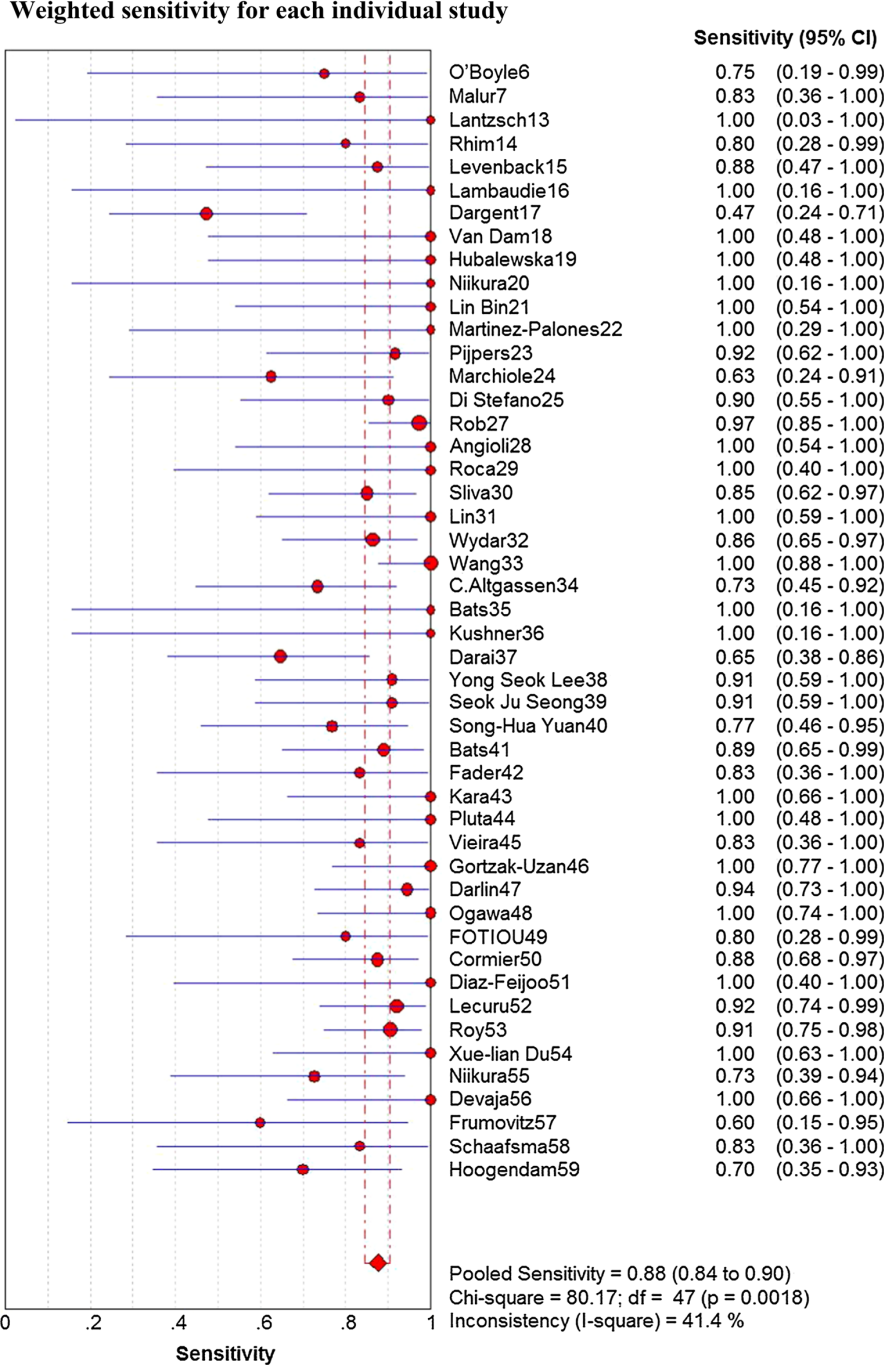
Weighted sensitivity for each individual study

Sensitivity for detecting lymph node metastases for all studies (*n* = 48) was 0.88 (95 % CI 0.84–0.90) (see Fig. 1), with heterogeneity [*χ* ^2^ = 80.17, degrees of freedom (*df*) = 47, *p* = 0.002]. Subgroup analysis for the three detection techniques revealed a homogeneous distribution if Tc99m only was used (*χ* ^2^ = 16.10, *df* = 9, *p* = 0.065). Studies including Tc99m colloid combined with blue dye were heterogeneous (*χ* ^2^ = 45.55, *df* = 28, *p* = 0.02). Also, studies in which blue dye was used were heterogeneous (*χ* ^2^ = 18.37, *df* = 7, *p* = 0.01). Tc99m used in a combination with blue dye yielded a pooled sensitivity of 0.88, but Tc99m use alone had the same pooled sensitivity of 0.87 blue dye used alone.

The pooled detection rate was 0.93 (95 % CI 0.92–0.94). No significant differences between tracers used were identified (detection rate: Tc99m 0.90 [95 % CI 0.87–0.93], blue dye 0.87 [95 % CI 0.84–0.90], combined Tc99m and blue dye 0.97 [95 % CI 0. 87–0.93]). The pooled detection rate of laparotomy versus laparoscopy versus robot-assisted surgery was 0.87 (95 % CI 0.83–0.91), 0.93 (95 % CI 0.90–0.96), and 0.92 (95 % CI 0.88–0.95), not significantly different (*p* > 0.05). Also, sensitivity did not significantly differ among laparotomy, 0.86 (95 % CI 0.80–0.90); laparoscopy, 0.90 (95 % CI 0.86–0.94); and robot-assisted surgery, 0.84 (95 % CI 0.72–0.92).

## Discussion

Detection of lymph node metastasis in early cervical cancer is crucial for guiding subsequent treatment. In this meta-analysis, we included 49 studies (2,476 various SLN procedures) and quantified pooled sensitivities and detection rates. Each procedure had high sensitivity for SLN detection (rate: 0.93 [95 % CI 0.92–0.94], sensitivity: 0.88 [95 % CI 0.84–0.91]), which was similar to those achieved in patients with breast cancer (sensitivity 0.91) [9]. Our work documents that SLN procedures are appropriately diagnostic for assessment of nodal metastases in patients with early stage cervical cancer.

FIGO clinical stages correlate with prognosis and anatomical extent of the disease but underestimates pathological extents of disease. Also, nodal involvement, an important independent prognostic factor, is often not incorporated into current FIGO classifications. SLN surgical procedures and detailed assessment should be standard care for early cervical cancer patients. We suggest that for every patient diagnosed with early cervical cancer without clinical evidence of lymph node involvement or metastatic disease, especially for those women desiring to retain fertility SLN procedures should be considered.

Better diagnostic technology and screening allows earlier detection of cervical cancer and ideally less metastases to lymph nodes. Currently, cervical cancer is diagnosed by endoscopic biopsy, followed by HPV testing and cervical cytology. Also, ultrasonography (US), computed tomography (CT), and magnetic resonance imaging (MRI) are routinely used for treatment planning, but their results do not alter the FIGO disease stages. Thus, morbidity and mortality due to unnecessary extensive resection, including para-aortic lymph node resection, will increase [8]. SLN offers prognosis and decreased need for extensive surgery.

Van de Lande and colleagues [70] published a systematic review using selected studies of the SLN procedure in patients with early cervical cancer. They compared techniques (blue dye, Tc99m, or the combined method) and report the highest success rate in terms of detection rate and sensitivity. A total of 23 studies (including 842 patients) were investigated, indicating the greatest SN detection rate (0.97) with the combined method which had a sensitivity of 0.92. These data are similar to our findings. Also, we compared studies using different surgery methods (laparotomy, laparoscopy, and robot-assisted surgery). SN detection rates and sensitivity of robot-assisted surgery were homogeneous, perhaps because they were so few (only four studies and these included laparotomy and laparoscopy).

Overall treatment decisions based on SLN assessment alone is still not optimal, because of wide variations in reported results due to patient population differences and unique methods used for SLN identification and definition. Our pooled estimates represent average overall effects, and improved methods of SLN assessment in early cervical cancer are needed, including tracer identification, prognostic value of micrometases and isolated tumor cells, patient selection, and surgical expertise.

Techniques used for lymph node assessment most probably influence SNL procedure sensitivity. Detailed assessment of SNLs should increase sensitivity more than single-section H&E assessment. Therefore, the most accurate study protocol should include the same pathological assessment for SLN and non-sentinel nodes. Variation in pathological techniques used across reports prevented a full analysis.

However, our review is limited. First, selective reporting bias occurs in many clinical fields, including diagnostic testing. The exclusion of conference abstracts, letters to the editor, and non-English-language studies may have led to publication bias. Second, a wide variation in patient selection, SLN procedures, surgeons, pathological techniques, and heterogeneity across institutions may have affected estimates of diagnostic accuracy. Also, due to the combination of heterogeneous results used for our analysis, our findings should be applied in an epidemiological sense and not to establish criteria for patient selection.

## Conclusions

Sentinel-lymph-node performs well diagnostically for assessing nodal metastases in patients with early stage cervical cancer. Large, multicenter studies with strict and homogenous patient selection and standardized SLN procedures and pathological techniques are required to investigate any added value of SLN in the future.

## References

[1] Jemal A, Bray F, Center MM, Ferlay J, Ward E, Forman D (2011). Global cancer statistics. CA Cancer J Clin.

[2] Siegel R, Naishadham D, Jemal A (2013). Cancer statistics, 2013. CA Cancer J Clin.

[3] Jemal A, Siegel R, Ward E, Murray T, Xu J, Thun MJ (2007). Cancer statistics, 2007. CA Cancer J Clin.

[4] Sakuragi N (2007). Up-to-date management of lymph node metastasis and the role of tailored lymphadenectomy in cervical cancer. Int J Clin Oncol.

[5] Darai E, Rouzier R, Ballester M, Barranger E, Coutant C (2008). Sentinel lymph node biopsy in gynaecological cancers: the importance of micrometastases in cervical cancer. Surg Oncol.

[6] Wang HY, Sun JM, Lu HF, Shi DR, Ou ZL, Ren YL (2006). Micrometastases detected by cytokeratin 19 expression in sentinel lymph nodes of patients with early-stage cervical cancer. Int J Gynecol Cancer.

[7] Martinez-Palones JM, Gil-Moreno A, Perez-Benavente MA, Roca I, Xercavins J (2004). Intraoperative sentinel node identification in early stage cervical cancer using a combination of radiolabeled albumin injection and isosulfan blue dye injection. Gynecol Oncol.

[8] Beesley V, Janda M, Eakin E, Obermair A, Battistutta D (2007). Lymphedema after gynecological cancer treatment. Cancer.

[9] Veronesi U, Paganelli G, Viale G, Luini A, Zurrida S, Galimberti V (2003). A randomized comparison of sentinel-node biopsy with routine axillary dissection in breast cancer. N Engl J Med.

[10] Morton DL, Thompson JF, Cochran AJ, Mozzillo N, Elashoff R, Essner R (2006). Sentinel-node biopsy or nodal observation in melanoma. N Engl J Med.

[11] Whiting P, Rutjes AWS, Reitsma JB, Bossuyt PMM, Kleijnen J (2003). The development of QUADAS: a tool for the quality assessment of studies of diagnostic accuracy included in systematic reviews. BMC Med Res Methodol.

[12] O’Boyle JD, Coleman RL, Bernstein SG, Lifshitz S, Muller CY, Miller DS (2000). Intraoperative lymphatic mapping in cervix cancer patients undergoing radical hysterectomy: a pilot study. Gynecol Oncol.

[13] Lantzsch T, Wolters M, Grimm J, Mende T, Buchmann J, Sliutz G (2001). Sentinel node procedure in Ib cervical cancer: a preliminary series. Br J Cancer.

[14] Malur S, Krause N, Kohler C, Schneider A (2001). Sentinel lymph node detection in patients with cervical cancer. Gynecol Oncol.

[15] Rhim CC, Park JS, Bae SN, Namkoong SE (2002). Sentinel node biopsy as an indicator for pelvic nodes dissection in early stage cervical cancer. J Korean Med Sci.

[16] Levenback C, Coleman RL, Burke TW, Lin WM, Erdman W, Deavers M (2002). Lymphatic mapping and sentinel node identification in patients with cervix cancer undergoing radical hysterectomy and pelvic lymphadenectomy. J Clin Oncol.

[17] Lambaudie E, Collinet P, Narducci F, Sonoda Y, Papageorgiou T, Carpentier P (2003). Laparoscopic identification of sentinel lymph nodes in early stage cervical cancer: prospective study using a combination of patent blue dye injection and technetium radiocolloid injection. Gynecol Oncol.

[18] Dargent D, Enria R (2003). Laparoscopic assessment of the sentinel lymph nodes in early cervical cancer. Technique—preliminary results and future developments. Crit Rev Oncol Hematol.

[19] van Dam PA, Hauspy J, Vanderheyden T, Sonnemans H, Spaepen A, Eggenstein G (2003). Intraoperative sentinel node identification with Technetium-99m-labeled nanocolloid in patients with cancer of the uterine cervix: a feasibility study. Int J Gynecol Cancer.

[20] Hubalewska A, Sowa-Staszczak A, Huszno B, Markocka A, Pitynski K, Basta A (2003). Use of Tc99m-nanocolloid for sentinel nodes identification in cervical cancer. Nucl Med Rev Cent East Eur.

[21] Niikura H, Okamura C, Akahira J, Takano T, Ito K, Okamura K (2004). Sentinel lymph node detection in early cervical cancer with combination 99mTc phytate and patent blue. Gynecol Oncol.

[22] Li B, Zhang WH, Liu L, Wu LY, Zhang R, Li N (2004). Sentinel lymph node identification in patients with early stage cervical cancer undergoing radical hysterectomy and pelvic lymphadenectomy. Chin Med J (Engl).

[23] Pijpers R, Buist MR, van Lingen A, Dijkstra J, van Diest PJ, Teule GJ (2004). The sentinel node in cervical cancer: scintigraphy and laparoscopic gamma probe-guided biopsy. Eur J Nucl Med Mol Imaging.

[24] Marchiolè P, Buénerd A, Scoazec J-Y, Dargent D, Mathevet P (2004). Sentinel lymph node biopsy is not accurate in predicting lymph node status for patients with cervical carcinoma. Cancer.

[25] Di Stefano AB, Acquaviva G, Garozzo G, Barbic M, Cvjeticanin B, Meglic L (2005). Lymph node mapping and sentinel node detection in patients with cervical carcinoma: a 2-year experience. Gynecol Oncol.

[26] Gil-Moreno A, Diaz-Feijoo B, Roca I, Puig O, Perez-Benavente MA, Aguilar I (2005). Total laparoscopic radical hysterectomy with intraoperative sentinel node identification in patients with early invasive cervical cancer. Gynecol Oncol.

[27] Rob L, Strnad P, Robova H, Charvat M, Pluta M, Schlegerova D (2005). Study of lymphatic mapping and sentinel node identification in early stage cervical cancer. Gynecol Oncol.

[28] Angioli R, Palaia I, Cipriani C, Muzii L, Calcagno M, Gullotta G (2005). Role of sentinel lymph node biopsy procedure in cervical cancer: a critical point of view. Gynecol Oncol.

[29] Roca I, Caresia AP, Gil-Moreno A, Pifarre P, Aguade-Bruix S, Castell-Conesa J (2005). Usefulness of sentinel lymph node detection in early stages of cervical cancer. Eur J Nucl Med Mol Imaging.

[30] Silva LB, Silva-Filho AL, Traiman P, Triginelli SA, de Lima CF, Siqueira CF (2005). Sentinel node detection in cervical cancer with (99m)Tc-phytate. Gynecol Oncol.

[31] Lin YS, Tzeng CC, Huang KF, Kang CY, Chia CC, Hsieh JF (2005). Sentinel node detection with radiocolloid lymphatic mapping in early invasive cervical cancer. Int J Gynecol Cancer.

[32] Wydra D, Sawicki S, Wojtylak S, Bandurski T, Emerich J (2006). Sentinel node identification in cervical cancer patients undergoing transperitoneal radical hysterectomy: a study of 100 cases. Int J Gynecol Cancer.

[33] Altgassen C, Paseka A, Urbanczyk H, Dimpfl T, Diedrich K, Dahmen G (2007). Dilution of dye improves parametrial SLN detection in patients with cervical cancer. Gynecol Oncol.

[34] Bats AS, Clement D, Larousserie F, Lefrere-Belda MA, Faraggi M, Froissart M (2007). Sentinel lymph node biopsy improves staging in early cervical cancer. Gynecol Oncol.

[35] Kushner DM, Connor JP, Wilson MA, Hafez GR, Chappell RJ, Stewart SL (2007). Laparoscopic sentinel lymph node mapping for cervix cancer—a detailed evaluation and time analysis. Gynecol Oncol.

[36] Darai E, Lavoue V, Rouzier R, Coutant C, Barranger E, Bats AS (2007). Contribution of the sentinel node procedure to tailoring the radicality of hysterectomy for cervical cancer. Gynecol Oncol.

[37] Lee YS, Rhim CC, Lee HN, Lee KH, Park JS, Namkoong SE (2007). HPV status in sentinel nodes might be a prognostic factor in cervical cancer. Gynecol Oncol.

[38] Seong SJ, Park H, Yang KM, Kim TJ, Lim KT, Shim JU (2007). Detection of sentinel lymph nodes in patients with early stage cervical cancer. J Korean Med Sci.

[39] Yuan SH, Xiong Y, Wei M, Yan XJ, Zhang HZ, Zeng YX (2007). Sentinel lymph node detection using methylene blue in patients with early stage cervical cancer. Gynecol Oncol.

[40] Bats AS, Lavoue V, Rouzier R, Coutant C, Kerrou K, Darai E (2008). Limits of day-before lymphoscintigraphy to localize sentinel nodes in women with cervical cancer. Ann Surg Oncol.

[41] Fader AN, Edwards RP, Cost M, Kanbour-Shakir A, Kelley JL, Schwartz B (2008). Sentinel lymph node biopsy in early-stage cervical cancer: utility of intraoperative versus postoperative assessment. Gynecol Oncol.

[42] Kara PP, Ayhan A, Caner B, Gultekin M, Ugur O, Bozkurt MF (2008). Sentinel lymph node detection in early stage cervical cancer: a prospective study comparing preoperative lymphoscintigraphy, intraoperative gamma probe, and blue dye. Ann Nucl Med.

[43] Vieira SC, Sousa RB, Tavares MB, Silva JB, Abreu BA, Santos LG (2009). Preoperative pelvic lymphoscintigraphy is of limited usefulness for sentinel lymph node detection in cervical cancer. Eur J Obstet Gynecol Reprod Biol.

[44] Pluta M, Rob L, Charvat M, Chmel R, Halaska M, Skapa P (2009). Less radical surgery than radical hysterectomy in early stage cervical cancer: a pilot study. Gynecol Oncol.

[45] Gortzak-Uzan L, Jimenez W, Nofech-Mozes S, Ismiil N, Khalifa MA, Dube V (2010). Sentinel lymph node biopsy vs. pelvic lymphadenectomy in early stage cervical cancer: is it time to change the gold standard?. Gynecol Oncol.

[46] Darlin L, Persson J, Bossmar T, Lindahl B, Kannisto P, Masback A (2010). The sentinel node concept in early cervical cancer performs well in tumors smaller than 2 cm. Gynecol Oncol.

[47] Ogawa S, Kobayashi H, Amada S, Yahata H, Sonoda K, Abe K (2010). Sentinel node detection with (99m)Tc phytate alone is satisfactory for cervical cancer patients undergoing radical hysterectomy and pelvic lymphadenectomy. Int J Clin Oncol..

[48] Cormier B, Diaz JP, Shih K, Sampson RM, Sonoda Y, Park KJ (2011). Establishing a sentinel lymph node mapping algorithm for the treatment of early cervical cancer. Gynecol Oncol.

[49] Fotiou S, Zarganis P, Vorgias G, Trivizaki E, Velentzas K, Akrivos T (2010). Clinical value of preoperative lymphoscintigraphy in patients with early cervical cancer considered for intraoperative lymphatic mapping. Anticancer Res.

[50] Diaz-Feijoo B, Perez-Benavente MA, Cabrera-Diaz S, Gil-Moreno A, Roca I, Franco-Camps S (2011). Change in clinical management of sentinel lymph node location in early stage cervical cancer: the role of SPECT/CT. Gynecol Oncol.

[51] Lecuru F, Mathevet P, Querleu D, Leblanc E, Morice P, Darai E (2011). Bilateral negative sentinel nodes accurately predict absence of lymph node metastasis in early cervical cancer: results of the SENTICOL study. J Clin Oncol.

[52] Roy M, Bouchard-Fortier G, Popa I, Gregoire J, Renaud MC, Tetu B (2011). Value of sentinel node mapping in cancer of the cervix. Gynecol Oncol.

[53] Du XL, Sheng XG, Jiang T, Li QS, Yu H, Pan CX (2011). Sentinel lymph node biopsy as guidance for radical trachelectomy in young patients with early stage cervical cancer. BMC Cancer.

[54] Niikura H, Okamoto S, Otsuki T, Yoshinaga K, Utsunomiya H, Nagase S (2012). Prospective study of sentinel lymph node biopsy without further pelvic lymphadenectomy in patients with sentinel lymph node-negative cervical cancer. Int J Gynecol Cancer.

[55] Frumovitz M, Euscher ED, Deavers MT, Soliman PT, Schmeler KM, Ramirez PT (2012). “Triple injection” lymphatic mapping technique to determine if parametrial nodes are the true sentinel lymph nodes in women with cervical cancer. Gynecol Oncol.

[56] Schaafsma BE, van der Vorst JR, Gaarenstroom KN, Peters AA, Verbeek FP, de Kroon CD (2012). Randomized comparison of near-infrared fluorescence lymphatic tracers for sentinel lymph node mapping of cervical cancer. Gynecol Oncol.

[57] Devaja O, Mehra G, Coutts M, Montalto SA, Donaldson J, Kodampur M (2012). A prospective single-center study of sentinel lymph node detection in cervical carcinoma: is there a place in clinical practice?. Int J Gynecol Cancer.

[58] Hoogendam JP, Hobbelink MG, Veldhuis WB, Verheijen RH, van Diest PJ, Zweemer RP (2013). Preoperative sentinel node mapping with (99m)Tc-nanocolloid SPECT-CT significantly reduces the intraoperative sentinel node retrieval time in robot assisted laparoscopic cervical cancer surgery. Gynecol Oncol.

[59] Verheijen RH, Pijpers R, van Diest PJ, Burger CW, Buist MR, Kenemans P (2000). Sentinel node detection in cervical cancer. Obstet Gynecol.

[60] Crane LM, Themelis G, Pleijhuis RG, Harlaar NJ, Sarantopoulos A, Arts HJ (2011). Intraoperative multispectral fluorescence imaging for the detection of the sentinel lymph node in cervical cancer: a novel concept. Mol Imaging Biol.

[61] Buist MR, Pijpers RJ, van Lingen A, van Diest PJ, Dijkstra J, Kenemans P (2003). Laparoscopic detection of sentinel lymph nodes followed by lymph node dissection in patients with early stage cervical cancer. Gynecol Oncol.

[62] Chung YA, Kim SH, Sohn HS, Chung SK, Rhim CC, Namkoong SE (2003). Usefulness of lymphoscintigraphy and intraoperative gamma probe detection in the identification of sentinel nodes in cervical cancer. Eur J Nucl Med Mol Imaging.

[63] Hauspy J, Beiner M, Harley I, Ehrlich L, Rasty G, Covens A (2007). Sentinel lymph nodes in early stage cervical cancer. Gynecol Oncol.

[64] Coutant C, Morel O, Delpech Y, Uzan S, Darai E, Barranger E (2007). Laparoscopic sentinel node biopsy in cervical cancer using a combined detection: 5-years experience. Ann Surg Oncol.

[65] Abu-Rustum NR, Neubauer N, Sonoda Y, Park KJ, Gemignani M, Alektiar KM (2008). Surgical and pathologic outcomes of fertility-sparing radical abdominal trachelectomy for FIGO stage IB1 cervical cancer. Gynecol Oncol.

[66] Zarganis P, Kondi-Pafiti A, Arapantoni-Dadioti P, Trivizaki E, Velentzas K, Vorgias G (2009). The sentinel node in cervical cancer patients: role of tumor size and invasion of lymphatic vascular space. In Vivo.

[67] Martinez A, Zerdoud S, Mery E, Bouissou E, Ferron G, Querleu D (2010). Hybrid imaging by SPECT/CT for sentinel lymph node detection in patients with cancer of the uterine cervix. Gynecol Oncol.

[68] Bats AS, Buenerd A, Querleu D, Leblanc E, Darai E, Morice P (2011). Diagnostic value of intraoperative examination of sentinel lymph node in early cervical cancer: a prospective, multicenter study. Gynecol Oncol.

[69] Chereau E, Feron JG, Ballester M, Coutant C, Bezu C, Rouzier R (2012). Contribution of pelvic and para-aortic lymphadenectomy with sentinel node biopsy in patients with IB2-IIB cervical cancer. Br J Cancer.

[70] van de Lande J, Torrenga B, Raijmakers PG, Hoekstra OS, van Baal MW, Brölmann HA (2007). Sentinel lymph node detection in early stage uterine cervix carcinoma: a systematic review. Gynecol Oncol.

